# Unraveling the occupational exposure to mycotoxins in a waste management setting: results from a case study in Norway

**DOI:** 10.3389/fpubh.2025.1536836

**Published:** 2025-02-13

**Authors:** Carla Martins, Carla Viegas, Elke Eriksen, Pål Graff, Anani Komlavi Afanou, Anne Straumfors, Magdalena Twarużek, Jan Grajewski, Robert Kosicki, Susana Viegas

**Affiliations:** ^1^NOVA National School of Public Health, Public Health Research Centre, Comprehensive Health Research Center, CHRC, REAL, CCAL, NOVA University Lisbon, Lisbon, Portugal; ^2^H&TRC–Health and Technology Research Center, ESTeSL–Escola Superior de Tecnologia da Saúde, Instituto Politécnico de Lisboa, Lisbon, Portugal; ^3^National Institute of Occupational Health (STAMI), Oslo, Norway; ^4^Department of Physiology and Toxicology, Faculty of Biological Sciences, Kazimierz Wielki University, Bydgoszcz, Poland

**Keywords:** mycotoxins, occupational hygiene, waste management, human biomonitoring, exposure assessment

## Abstract

**Introduction:**

Waste management represents an occupational setting where fungi are significant contaminants. This study aimed to assess the exposure of waste workers to mycotoxins through a human biomonitoring study.

**Methods:**

A total of 33 workers and 19 controls provided spot urine samples to determine 10 mycotoxins’ urinary biomarkers using liquid chromatography coupled with mass spectrometry. Risk characterization was performed using hazard quotient and margin of exposure assessments.

**Results:**

The results indicated that workers were exposed to six out of the 10 mycotoxins tested, with the following detection rates: deoxynivalenol (91%, 30/33), ochratoxin A (33%, 11/33), zearalenone (17%, 5/33), α-zearalenol (12%, 4/33), β-zearalenol (12%, 4/33), and HT-2 toxin (3%, 1/33). Within controls and outwith controls, were exposed to 5/10 and 2/10 mycotoxins, respectively. All participants exhibited hazard quotients for deoxynivalenol and zearalenone below one, indicating that the exposure is unlikely to pose a health risk. However, when considering the margin of exposure determined for ochratoxin A, 18% of the total participants presented results below 200 for non-neoplastic effects, and 100% of the total participants presented values below 10,000 for neoplastic effects, suggesting potential health concerns that require further assessment.

**Discussion:**

This study highlights the need for future research on occupational exposure to mycotoxins in waste management settings.

## 1 Introduction

Mycotoxins are secondary metabolites produced by various fungi on diverse human crops, explaining the commonly reported presence in food and feedstuffs, representing a major threat to human and animal health due to different toxic effects, such as cancer, mutagenicity, nephrotoxicity, estrogenicity, and other effects (1–3). Mycotoxins are non-volatile substances, and they bind to particulate matter. Therefore, factors that facilitate the release and resuspension of particles will contribute to increased exposure to airborne mycotoxin exposure (4). The smaller particles are distributed through diffusion to the lung and bronchioles and, from there, reach the bloodstream (5). In addition to inhalation, dermal exposure is also a possibility, with existing evidence regarding the penetration of skin by mycotoxins (4, 6).

Although the consumption of contaminated food is the primary source of human exposure to mycotoxins, occupational environments may also present risks through additional exposure routes, such as inhalation and dermal absorption (7–9). Consequently, in different working routine activities (storage work, loading, handling, or milling contaminated materials (e.g., grain and feed) in different types of industries (e.g., brewing, bakeries), and others such as caring for animals in animal husbandry settings), workers can be exposed to high amounts of organic dust that contains multiple fungal species as well as mycotoxins (8, 10–13). Mycotoxins, such as aflatoxins, ochratoxin A, and trichothecenes, have previously been identified as occupational hazards due to their toxic effects, including immunosuppression, nephrotoxicity, and carcinogenicity with chronic exposure. They have been detected present in several workplaces (4–6, 14). Recent research has revealed relatively high levels of fungal spores and fragments with high inflammatory potential in contemporary waste-sorting plants (15–17). Occupational exposure to organic dust containing high levels of fungi is a significant health concern for workers involved in waste handling and sorting, where the decomposition of organic material contained in residual waste creates an ideal environment for fungal proliferation.

Exposure to airborne fungi may be especially high during certain work tasks, such as cleaning with compressed air, which aerosolizes settled dust, and manual sorting of paper and cardboard (16). *Aspergillus* spp. and *Penicillium* spp. have been identified to be among the most prevalent fungi encountered in the waste sorting industry (17). Given the potential for chronic work exposure and a potential for developing exposure-related health effects, it is critical to conduct comprehensive risk assessments and to mitigate the risks associated with exposure to fungal and mycotoxin contamination in waste management environments. This study aimed to assess the exposure of waste management workers to mycotoxins at automated and manual plants dedicated to residual waste in Norway.

## 2 Materials and methods

### 2.1 The occupational setting, participants, and sampling

The study was conducted in four waste management companies located in Norway, with sorted waste volumes ranging from 50 to 347 k tons per year. The facilities were dedicated to waste sorting in two different systems: (i) manual plants, dedicated to waste from housing collectives and local businesses, with plastic and paper/cardboard manually sorted and residual waste sorted by workers; and (ii) automated plants, where unsorted residual waste from domestic homes was received, and sorting was achieved by modern and fully automated waste sorting lines.

Regarding the participants, three different groups were considered: (i) workers directly involved in waste sorting, (ii) workers from waste plants performing administrative tasks (within controls), and (iii) individuals from the general population (outwith controls). Of the 93 eligible workers, 50 agreed to participate (54% participation rate), and 40 provided a spot urine sample (43% sampling rate). Regarding the control group invited for participation, 14 of 17 accepted to participate (rate of participation: 82%). The outwith control group (*n* = 5) completed the control group, thus being a total of 19 participants. Workers did not work on weekends. A sampling of urine was performed at a one-time point (known as spot sampling) on Wednesdays, the third day of the sampling campaign, and the third day of the work week. Participants provided a spot urine sample in the afternoon, close to the end of the workday. Sampling campaigns were conducted during summer and autumn. Samples were stored frozen at −18°C until and during shipment. Samples from seven participants were not analyzed due to transportation problems. Therefore, the final number of workers in the present study is 33.

### 2.2 Ethical considerations

This study was conducted according to the Helsinki Declaration and Oviedo Convention, and all data were stored and analyzed in accordance with the Portuguese General Data Protection Regulation (GDPR) law n° 58/2019. The study was approved by the Regional Committees for Medical Research Ethics Southeast Norway, REK South East (ref. no. 34312). Participation in the study was voluntary, and informed consent was obtained prior to participation.

### 2.3 Questionnaires

Sociodemographic and contextual data were collected through a questionnaire adapted from the NOSQ-2002 Nordic Occupational Skin Questionnaire (18) in combination with questions that surveyed personal data (e.g., sex, age, and smoking habits), work-related data (e.g., work hours per day/week, time of employment), and health-related data on symptom frequencies with a focus on airway symptoms, as well as symptoms of the gastrointestinal tract and skin. All data collected was based on self-reporting. The questionnaire was previously used in a study recently published (16).

### 2.4 Analytical determination of mycotoxins

The mycotoxins’ biomarkers [deoxynivalenol (DON), de epoxy-deoxynivalenol (DOM1), zearalenone (ZEN), alpha-zearalenone (α-ZOL), beta-zearalenone (β-ZOL), alpha-zearalanol (α-ZAL), beta-zearalanol (β-ZAL), T2 Toxin (T2), HT2 Toxin (HT2), ochratoxin A (OTA)] were determined in urine samples, according to the following procedures.

#### 2.4.1 Chemicals

Standard solutions of DON, DOM1, ZEN, α-ZOL, β-ZOL, α-ZAL, β-ZAL, T2, HT2, and OTA, along with internal isotope-labeled standards U-[^13^C_15_]-DON, U-[^13^C_18_]-ZEN, U-[^13^C_24_]-T-2, U-[^13^C_22_]-HT-2 and U-[^13^C_20_]-OTA, were acquired from Romer Labs Diagnostic (Tulln, Austria). The creatinine standard and trifluoroacetic acid were obtained from Sigma–Aldrich (Darmstadt, Germany). Acetonitrile (gradient grade), methanol (LC–MS grade), ammonium acetate, and acetic acid were purchased from Merck (Darmstadt, Germany). Immunoaffinity columns Ochraprep® and DZT MS-PREP®, along with phosphate-buffered saline tablets, were provided by R-Biopharm Rhone (Glasgow, UK). A β-glucuronidase/arylsulfatase solution derived from *Helix pomatia* (with specific activities of 5.5 U/ml and 2.6 U/ml at +38°C, respectively) and potassium acetate hydrolysis buffer (pH 5) was purchased from Sigma-Aldrich (Darmstadt, Germany). Deionized water was purified using a Simplicity UV water purification system (Millipore, USA).

#### 2.4.2 Sample preparation

For mycotoxin measurement, urine samples were centrifuged at 7,000 rpm for 10 min after thawing. A volume of 2.5 ml of the supernatant was then combined with 25 μl of a beta-glucuronidase/arylsulfatase enzyme solution and 0.25 ml of a potassium acetate hydrolysis buffer. The mixture was incubated overnight at 37°C. The following day, the samples were diluted with 5 ml of phosphate-buffered saline and 50 μl of a mixture of internal standards. The diluted samples were mixed using a rotary shaker and then applied quantitatively to Ochraprep® and DZT MS-PREP® immunoaffinity columns arranged in series with SPE tube adaptors. The columns were washed with 10 ml of distilled water, and mycotoxins were eluted using 3 ml of methanol. The eluted extracts were evaporated to dryness under a stream of nitrogen at 45°C. The resulting residue was reconstituted in 500 μl of a methanol/water mixture (1:4, v/v) and transferred to autosampler vials for further analysis.

For creatinine measurement, urine sample preparation followed the protocol described by Warth et al. (19). In brief, the urine samples were centrifuged at 7,000 rpm for 10 min. Subsequently, 10 μl of the supernatant was diluted with a 1:10000 water/acetonitrile mixture (9:1, v/v). The diluted samples were then centrifuged again at 14,000 rpm for 10 min before being transferred to autosampler vials for analysis.

#### 2.4.3 Chromatographic analysis

The analysis of mycotoxins was conducted using a Nexera^â^ high-performance liquid chromatography (HPLC) system (Shimadzu, Kyoto, Japan) coupled with a QTRAP 5500 mass spectrometer (Sciex^â^, Framingham, MA, USA). The separation was carried out on a Kinetex C18 column (100 × 2.1 mm, 2.6 μm) equipped with a guard column from Phenomenex (Torrance, USA) at a maintained column temperature of 40°C. The chromatographic separation utilized gradient elution with a flow rate of 0.3 ml/min. The mobile phase A comprised water with 5 mM ammonium acetate and 0.1% acetic acid, while mobile phase B consisted of methanol with 5 mM ammonium acetate and 0.1% acetic acid. The gradient elution program was set as follows: 0 min, 15% B; 14.2 min, 68% B; 14.5 min, 95% B; 17.0 min, 95% B; 17.1 min, 15% B; 22.0 min, 15% B. A sample injection volume of 10 μl was used. The mass spectrometer was equipped with an electrospray ionization (ESI) interface and operated in both negative and positive ion modes with voltages of −4,500 V and +4,500 V, respectively, in the scheduled multiple reaction monitoring (sMRM) mode. A diverter valve was utilized to exclude the initial 1.5 min and the final 8.2 min of each chromatographic run to prevent contamination of the mass spectrometer by unwanted polar compounds. The source/gas conditions were optimized as follows: the curtain gas (CUR) was set at 30 psi, the source temperature (TEM) at 550°C, both the nebulizer gas (GS1) and heater gas (GS2) at 80 psi, and the collision gas (CAD) at medium. The optimization of compound-dependent parameters, including the declustering potential (DP), collision energy (CE), and collision cell exit potential (CXP), was achieved through flow injection analysis (Table 1). Data acquisition and processing were performed using the Analyst 1.6.2 software.

**Table 1:** Parameters optimized for the quantification of mycotoxins using electrospray ionization tandem mass spectrometry (ESI-MS/MS).

Compound	Q1 [m/z]^a^	Q3 [m/z]^a^	Retention time [min]^b^	DP [V]	CE [V]	CXP [V]
^13^C_15_ DON	370.2	279.3	2.1	−70	−22	−7
^13^C_22_ HT-2	464.1	278.1	10.9	76	19	16
^13^C_20_ OTA	424.1	250.1	11.8	131	33	16
^13^C_24_ T-2	508.2	322.1	12.4	86	19	20
^13^C_18_ ZEN	335.3	140.0	13.3	−125	−42	−19
α-ZAL	321.2	277.2	12.8	−115	−32	−13
	321.2	303.2	12.8	−115	−30	−15
α-ZOL	319.2	160.0	13.1	−115	−44	−13
	319.2	130.0	13.1	−115	−50	−20
β-ZAL	321.2	277.2	11.4	−115	−32	−13
	321.2	303.2	11.4	−115	−30	−15
β-ZOL	319.2	160.0	11.8	−115	−44	−13
	319.2	130.0	11.8	−115	−50	−20
DOM1	339.1	59.1	3.4	−70	−20	−9
	339.1	249.0	3.4	−70	−18	−17
DON	355.1	265.2	2.1	−75	−22	−13
	355.1	59.2	2.1	−75	−42	−9
HT2	442.1	263.1	10.9	71	19	14
	442.1	215.1	10.9	71	19	12
OTA	404.0	239.0	11.8	121	33	14
	404.0	102.0	11.8	121	87	14
T2	484.2	305.2	12.4	86	19	6
	484.2	215.1	12.4	86	25	12
ZEN	317.1	131.1	13.3	−110	−42	−8
	317.1	175.0	13.3	−110	−34	−13

^13^C_15_ DON, isotope-labelled (^13^C_15_) deoxynivalenol; ^13^C_24_ T-2, isotope-labelled (^13^C_24_) T-2 toxin; ^13^C_22_ HT-2, isotope-labelled (^13^C_22_) HT-2 toxin; ^13^C_18_ ZEN, isotope-labelled (^13^C_15_) zearalenone; ^13^C_20_ OTA, isotope-labelled (^13^C_20_) ochratoxin A; DON, deoxynivalenol; DOM1, deepoxy-deoxynivalenol; ZEN, zearalenone; α-ZOL, alpha-zearalenol; β-ZOL, beta-zearalenol; α-ZAL, alpha-zearalanol; β-ZAL, beta-zearalanol; T2, T-2 toxin; HT2, HT-2 toxin; OTA, ochratoxin A; Q1, First Quadrupole; Q3, Third Quadrupole; DP, Declustering Potential; CE, Collision Energy; CXP=Collision Cell Exit Potential.

a MS transitions are given for the quantifier ion on top and the qualifier ion below.

b With expected retention times, as they were placed in the Analyst software.

#### 2.4.4 Method validation

The method’s validation involved assessing the limits of detection (LOD) and quantification (LOQ), the applicable working ranges, recovery rates, precision (RSD), and matrix effects. The LOD and LOQ were determined using a signal-to-noise ratio of 3 and 10, respectively, employing a script within Analyst software. Calibration curves, consisting of at least six points, were generated for each analyte to establish the working range. Recovery rates were evaluated by spiking urine samples devoid of mycotoxins at three distinct concentration levels. Precision was ascertained through three independent replicates for each concentration level. Matrix effects, quantified as signal suppression or enhancement (SSE), were assessed by comparing the slopes of calibration curves prepared with matrix-matched and pure solvent solutions according to the equation:

$$SSE\;\left[\%\right]=100^{*}\;{\mathrm{slope}}_{\mathrm{matrix}-\mathrm{matched calibration}}/{\mathrm{slope}}_{\mathrm{pure solvent calibration}}$$

### 2.5 Risk characterization

To perform risk characterization, mycotoxin concentrations in urine were compared with different health-based guidance values, thus determining the Hazard Quotient (HQ) and the Margin of Exposure (MoE). Results of HQ above one (>1) indicate a potential health concern, and the magnitude of MoE indicates the risk level as well. Regarding OTA, EFSA concluded that an MoE above 200 and 10,000 was of low concern for public health, for non-neoplastic and neoplastic effects, respectively (20). Probable Daily Intake (PDI) for each mycotoxin was determined through reverse dosimetry calculation to convert the urinary mycotoxin concentrations into intake levels, expressed as μg/kg bw/day. The deterministic method of intake mass balance was applied, considering the concentration of the biomarker in urine (μg/L), the urinary volume produced in 24 h (L), the body weight (kg), and the excretion rate for each mycotoxin (%). Urinary volume for 24 h was derived from body weight considering 20 ml/kg for participants, which is in line with previous HBM4EU estimations (21). All the data regarding the participants (body weight, urinary biomarker concentration, and urinary volume in 24 h) were considered at an individual level. Excretion rates considered were 64.0% for DON (22), 9.6% for ZEN (19), and 2.5% for OTA (23). Exposure results were compared with the Tolerable Daily Intake (TDI) for DON (1.0 μg/kg bw/day) and for ZEN (0.250 μg/kg bw/day) and with the Benchmark Dose (lower confidence limit) (BMDL) for OTA for non-neoplastic effects (4.73 μg/kg bw/day) and neoplastic effects (14.5 μg/kg bw/day) (20, 24, 25). Since there is a Human Biomonitoring Guidance Value (HBM-GV) for DON in urine (23 μg/L DON in urine) (26), urinary concentrations of DON at the individual level were also compared with this HBM-GV for determining the HQ.

### 2.6 Statistical analysis

The results of the biomarkers of exposure were presented as volume-weighted concentrations (μg/L) and as creatinine-adjusted concentrations (μg biomarker/g crea). Samples were considered positive for exposure to mycotoxins if at least one biomarker of exposure was determined in concentrations above the respective LOQ. Regarding the treatment of left-censored data for statistical analysis, due to the reduced number of observations in each group (controls and workers), a conservative approach was chosen, and the results of biomarkers below the LOD and LOQ were replaced by ½ LOD and ½ LOQ (middle-bound approach), respectively (27). Similarly to previous HBM studies, biomarker results with a frequency of quantification below 10% were not treated for left-censored data, and the results were presented only for positive samples (28). Descriptive statistics (frequencies, mean, median, and range) were performed with the data set. The normality of distributions of urinary biomarker variables was checked with the Shapiro–Wilk test. Since the data did not follow a normal distribution, non-parametric tests were used for further statistical analysis. Differences in concentrations of biomarkers, with a frequency of detection above 10%, between workers and the control group were analyzed with the Mann–Whitney U test, and differences between workers, within controls, and outwith control groups were analyzed with the Kruskal–Wallis test (28).

## 3 Results and discussion

### 3.1 Participants

Participants were classified into three groups: workers (*n* = 33), within controls (*n* = 14), and outwith controls (*n* = 5), and their characteristics are described in Table 2.

**Table 2:** Characteristics of participants (workers, within controls, and outwith controls).

	Participants (*n* = 33)	Within controls (*n* = 14)	Outwith controls (*n* = 5)
Age, years (Mean ± SD)	37.1 ± 10.8	39.5 ± 8.40	35.2 ± 4.10
Weight, Kg (Mean ± SD)	84.1 ± 15.1	83.1 ± 15.0	64.2 ± 10.20
Height, m (Mean ± SD)	1.77 ± 0.06	1.78 ± 0.09	1.65 ± 0.06
Sex	Woman, n = 4	Woman, n = 4	Woman, n = 5
	Man, n = 29	Man, n = 10	Man, n = 0

SD, Standard deviation.

### 3.2 Urinary biomarkers of exposure

The results of the performance characteristics of the LC–MS/MS method, which allowed the identification and quantification of biomarkers of exposure to mycotoxins, are presented in Table 3. All analytes presented good linear responses, recoveries ranged from 90.3 to 144.0%, and the maximum RSD was 8.9%. Thus, the analytical method was considered fit for the purpose.

**Table 3:** Validation parameters.

Analyte	LOD	LOQ	Working range	Low concentrations			Medium concentrations			High concentrations			Average recoveryn = 9	SSE with IS	SSE without IS
				Conc	Recoveryn = 3	RSD	Conc	Recoveryn = 3	RSD	Conc	Recoveryn = 3	RSD			
	μg/L	μg/L	μg/L	μg/L	[%]	[%]	μg/L	[%]	[%]	μg/L	[%]	[%]	[%]	[%]	[%]
α-ZAL	0.009	0.028	0.028–10	1.0	94.0	3.6	2.5	100.1	2.3	5.0	98.5	1.2	97.5	103	129
α-ZOL	0.007	0.025	0.025–10	1.0	85.1	4.5	2.5	93.2	1.3	5.0	92.5	1.8	90.3	99	124
β-ZAL	0.006	0.019	0.019–10	1.0	115.6	4.6	2.5	127.2	3.1	5.0	123.5	4.3	122.1	129	162
β-ZOL	0.008	0.026	0.026–10	1.0	137.6	5.3	2.5	150.0	2.8	5.0	144.4	1.7	144.0	148	186
DOM1	0.049	0.165	0.165–100	10	96.0	1.3	25	100.0	1.8	50	98.4	0.7	98.1	103	103
DON	0.043	0.143	0.143–100	10	106.2	5.0	25	100.0	1.6	50	100.2	0.9	102.1	102	102
HT2	0.008	0.027	0.027–40	4.0	98.5	0.9	10	101.1	1.4	20	100.1	2.1	99.9	104	105
OTA	0.004	0.014	0.014–2.0	0.2	101.1	8.9	0.5	97.5	3.4	1	94.3	2.7	97.6	102	115
T2	0.003	0.009	0.009–20	2.0	99.8	1.6	5.0	100.8	1.2	10	101.5	1.1	100.7	105	114
ZEN	0.004	0.014	0.014–10	1.0	96.7	4.7	2.5	102.1	1.2	5.0	101.0	1.4	99.9	107	135

DON, deoxynivalenol; DOM1, deepoxy-deoxynivalenol; ZEN, zearalenone; α-ZOL, alpha-zearalenol; β-ZOL, beta-zearalenol; α-ZAL, alpha-zearalanol; β-ZAL, beta-zearalanol; T2, T-2 toxin; HT2, HT-2 toxin; OTA, ochratoxin A; LOD, limit of detection; LOQ, limit of quantification; SSE, signal suppression or enhancement (with or without internal standard) is calculated by the ratio of the matrix-matched and the pure solvent calibration curve; RSD, Relative Standard Deviation.

All urine samples were negative for β-ZAL, α-ZAL, and T-2 toxin. DOM1 was detected in one urine sample but not quantified (<LOQ). Workers were exposed to 6/10 mycotoxins, with urine samples being positive for DON (91%, 30/33), OTA (33%, 11/33), ZEN (17%, 5/33), α-ZOL (12%, 4/33), β-ZOL (12%, 4/33), and HT-2 (3%, 1/33). Within controls were exposed to 5/10 mycotoxins, with urine samples being positive for DON (93%, 13/14), ZEN (29%, 4/14), OTA (21%, 3/14), α-ZOL (14%, 2/14), and HT2 (14%, 2/14). Outwith controls were exposed to 2/10 mycotoxins, with urine samples being positive for DON (80%, 4/5) and OTA (20%, 1/5). Results are presented in Table 4 as volume-weighted concentrations (μg/L) and as creatinine-adjusted concentrations (μg biomarker/g crea) for biomarkers.

**Table 4:** Concentrations of mycotoxins’ urinary biomarkers in workers and controls.

		Workers (*n* = 33)			Within controls (*n* = 14)			Outwith controls (*n* = 5)		
Biomarker	Units	Mean	Median	Range	Mean	Median	Range	Mean	Median	Range
DON	μg/L	1.06	0.94	LOQ-3.10	0.87	0.34	LOD-4.76	0.19	0.21	LOD-0.32
	μg/g crea	0.66	0.55	LOQ-2.39	0.89	0.55	LOD-2.98	0.45	0.52	LOD-0.84
ZEN	μg/L	0.013	0.002	LOD–0.106	0.010	0.007	LOD-0.048	ND	ND	ND
	μg/g crea	0.008	0.002	LOD-0.053	0.011	0.005	LOD-0.034	ND	ND	ND
α-ZOL	μg/L	0.011	0.004	LOD–0.099	0.010	0.004	LOD-0.041	ND	ND	ND
	μg/g crea	0.007	0.002	LOD-0.050	0.011	0.008	LOD-0.032	ND	ND	ND
OTA	μg/L	0.015	0.007	LOD-0.074	0.011	0.010	LOD-0.039	0.040^*^	-	-
	μg/g crea	0.009	0.004	LOD-0.037	0.009	0.007	LOD-0.017	0.037^*^	-	-
β-ZOL	μg/L	0.036	0.036	LOD-0.044	ND	ND	ND	ND	ND	ND
	μg/g crea	0.022	0.022	LOD-0.027	ND	ND	ND	ND	ND	ND
HT2	μg/L	0.030^*^	-	-	0.049^**^	ND	ND	ND	ND	ND
	μg/g crea	0.024^*^	-	-	0.040^**^	ND	ND	ND	ND	ND

DON, Deoxynivalenol; ZEN, Zearalenone; α-ZOL, alpha-zearalenone; OTA, Ochratoxin A; HT2, HT-2 toxin; SD, Standard Deviation; P95, Percentile 95; ND, Not Detected.

* Result of one urine sample.

** Result of two urine samples.

DON was the predominant mycotoxin detected in urine samples, with the highest concentrations in both workers and controls (within and outwith controls), followed by OTA, ZEN, and metabolites. β-ZOL was quantified in 4/33 workers (8%) in concentrations ranging from 0.028 to 0.044 μg/L. HT2 was quantified in one worker with a concentration of 0.030 μg/L and in two controls with concentrations of 0.028 μg/L and 0.069 μg/L.

Regarding the exposure to DON, ZEN, α-ZOL, and OTA, there were no statistically significant differences between the workers and controls when considering the control group as a whole (*p* > 0.05). However, when comparing the group of workers (*n* = 33) with the outwith control group (*n* = 5), significant differences were found (*p* = 0.032). The absence of differences between workers and within the control group may be explained by the hand contamination detected, with the presence of the same fungi, *Penicillium* sp. and *Cladosporium* sp. (29). Furthermore, concentrations of airborne microorganisms generally increase in areas where waste with potentially high microbial content is handled, meaning that within the control group, participants are, to some extent, exposed to higher levels of mycotoxins than the general population (17). Workers presented an exposure to DON approximately five times higher than out with controls. Nevertheless, it should be noted that these groups present a different number of participants (*n* = 33 vs. *n* = 5), which could result in some unbalanced comparison; therefore, these differences should be considered carefully. Potential differences in levels of exposure between seasons (autumn vs. summer) and type of plant (automatic vs. manual) were also assessed, but no statistically significant differences were found. Results obtained for urinary biomarkers partially agree with previous studies conducted in the same waste management facilities (16, 17); identifying OTA in urine samples agrees well with the detection of *Aspergillus* spp. and *Penicillium* spp. detected in industrial hygiene samples collected simultaneously (29). However, no *Fusarium* spp. fungi were found to corroborate with ZEN and DON results in biological samples.

**Figure 1:**
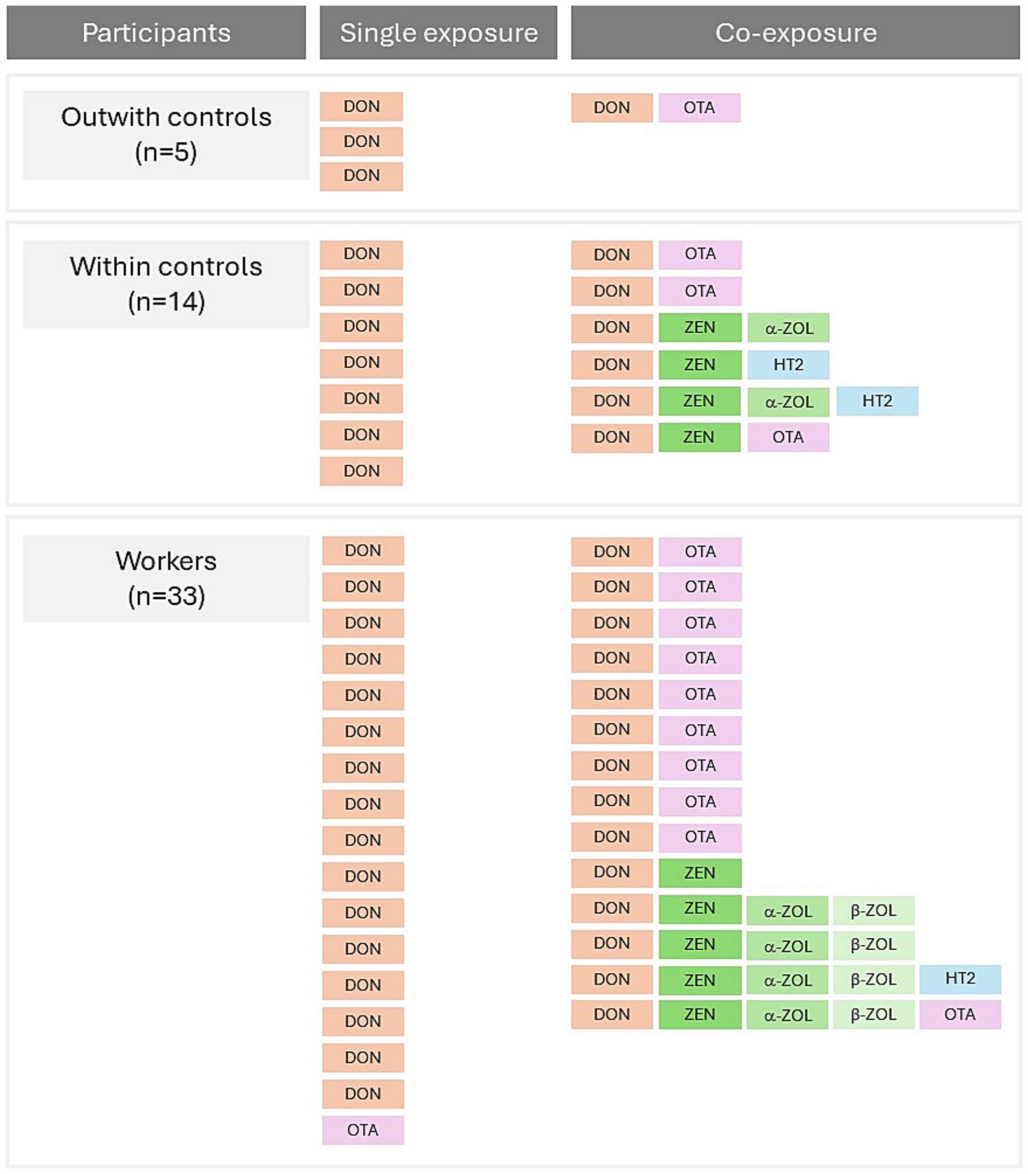
Simultaneous exposure to mycotoxins (DON, ZEN, α-ZOL, β-ZOL, HT2, OTA) for the three groups: workers, within controls, and outwith controls.

Regarding the exposure pattern, participants showed some differences in the number and type of urinary biomarkers detected simultaneously. Urine samples presented mixtures from two to five biomarkers (14/33, 42%), two to four biomarkers (6/14, 42%), and two biomarkers (1/5, 20%), for the workers, within controls, and outwith controls, respectively (Figure 1). Waste sorting workers were exposed to more than one mycotoxin in a similar proportion of the participants within the control group. The participants of the general population (outwith controls) had a reduced frequency of co-exposure; however, they did not have statistical significance from the remaining groups. The influence of the occupational context of waste management in the pattern of exposure to mycotoxins, particularly the co-exposure, requires further investigation. Nevertheless, these results agree well with other studies that previously reported co-exposure to mycotoxins as the regular pattern of exposure in occupational contexts (30).

The results of this study align with findings from previous studies in occupational settings. Viegas et al. reported exposure of bakery workers to OTA, DON, citrinin, enniatin B, and aflatoxin M1 in low concentrations for all mycotoxins analyzed (31). Ndaw et al. reported exposure of grain workers to dust generated when handling grain to DON, ZEN, OTA, α-ZOL, aflatoxin B_1_, and aflatoxin M_1_ (32). The concentrations reported in grain workers were, however, higher than the ones reported in the present study. In a similar setting, exposure to mycotoxins of mill workers in Germany was assessed, and DON, OTA, ZEN, and citrinin were detected in almost all urine samples, again in higher concentrations (33). In a different setting, animal production, co-exposure was reported as well: two different combinations of three mycotoxins (DON, aflatoxins, and OTA; aflatoxins, OTA, and citrinin) and the most common identified being OTA and DON (12). Regarding levels of exposure to OTA, no direct comparison with the present study is possible due to differences in instrumental limits (LOD and LOQ).

### 3.3 Risk characterization

**Figure 2:**
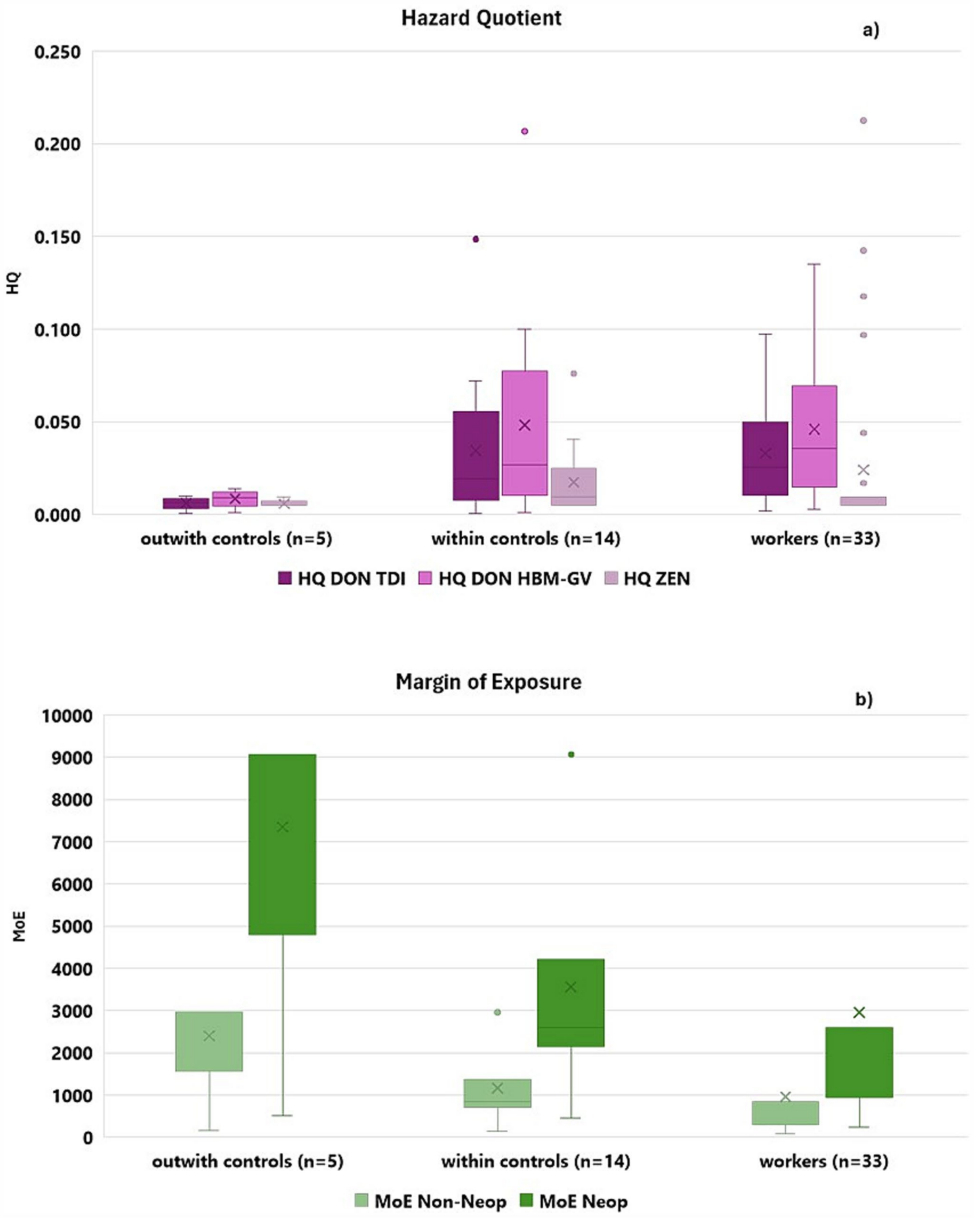
Hazard quotient (HQ; **A**) estimated for DON and ZEN and Margin of Exposure (MoE; **B**) for non-neoplastic effects and neoplastic effects estimated for OTA, in waste sorting workers, within control and outwith control groups. For DON, HQ was calculated using the TDI and the HBM-GV. Results below 1 for HQ (HQ < 1) do not represent a potential health concern from a public health perspective. Results above 200 and 10,000 for MoE regarding non-neoplastic (MoE > 200) and neoplastic effects (MoE > 10,000) do not represent a potential health concern from a public health perspective.

The risk characterization was achieved by determining the HQ and MoE, where appropriate. Results of HQ (DON and ZEN) and MoE (OTA) are presented in Figures 2A,B. Detailed data is available in Supplementary Figure S1.

Among all participants, the HQ for DON ranged from 0 to 0.207 when compared to the HBM-GV and from 0 to 0.149 when compared to the TDI. Since all HQ values were far below one, the exposure did not represent a potential health concern from a public health perspective, either for workers or controls. The two approaches (HQ using TDI or HBM-GV) presented similar results, which may be explained by the fact that the derivation of DON HBM-GV was based on the established TDI (26). Significant differences were found between workers and outwith controls for HQ based on TDI (*p* = 0.025) and HBM-GV (*p* = 0.031). Results obtained for HQ ZEN ranged from 0 to 0.212, and no statistically significant differences were found between the three groups of participants (*p* > 0.05).

When considering the MoE determined for OTA, seven participants presented results below 200 for non-neoplastic effects, and all participants presented results below 10,000 for neoplastic effects, which may represent a potential health concern and require further assessment. However, uncertainties surrounding the OTA excretion rate, as highlighted by other authors, should be considered. The complex relationship between OTA excretion in urine and OTA intake—due to factors like plasma protein binding, enterohepatic recirculation, and transport proteins—can affect the estimated PDI, warranting cautious interpretation of results (23, 34). Future studies may benefit from the simultaneous determination of biomarkers for both exposure and effect, which would enable the assessment of early effects on the kidney (35).

Given that simultaneous exposure to several mycotoxins is common in occupational settings and the general population, these results should be further investigated in future studies. The control group included participants working in waste management but not performing tasks directly involving waste and participants from the general population to ensure that occupational exposure did not influence the results obtained for urinary biomarkers. As in other occupational exposure contexts, the number of participants in the study is low. Therefore, results should be carefully considered when extrapolating to other contexts or population groups. Risk assessment is usually performed from a single substance perspective, and the obtained results emphasized the need to consider possible interaction effects (additive or synergistic) when evaluating potential health risks for better management in public health and environmental protection from hazardous chemical mixtures (36, 37). In this study, data on food consumption were not collected. Exposure of the Norwegian general population, especially to DON, is frequent due to the contamination of cereal commodities (38, 39). Participants included in the outwith control group confirm this exposure pattern by presenting concentrations of urinary DON above the LOD (4/5). The absence of this information is a limitation of the present study and may hamper more detailed conclusions about the exposure pattern.

Apart from OTA, results obtained for risk characterization for the remaining mycotoxins do not indicate a potential health concern. Nevertheless, it is important to guarantee that exposure to mycotoxins is as reduced as possible. Several measures are therefore recommended from an occupational hygiene perspective. It is important to provide training sessions for workers, explaining the use of personal protective equipment (e.g., respiratory devices, gloves) and promoting awareness of the importance of its use (16, 17). This training should reinforce the importance of hygiene measures before and after working hours, before breaks, and in accessing other areas (e.g., canteens, restrooms, offices) (29, 40). In workspaces, it is important to delineate clean and dirty areas clearly, utilize local exhaust ventilation, confine tasks that are known to produce dust, and establish cleaning and maintenance programs (16, 17). The results of studies such as this one should be considered important contributions and integrated into the development of health surveillance programs.

## 4 Conclusion

The present study concluded that waste management is an occupational setting characterized by exposure to various mycotoxins, including DON, ZEN, OTA, HT2, α-ZOL, and β-ZOL. Among these, DON was the most frequently detected mycotoxin in urine samples and was quantified at higher concentrations compared to others. Exposure levels were consistent among workers and higher compared to controls. Notably, 42% of workers, 42% of control within the waste management setting, and 20% of outwith controls were exposed to several mycotoxins simultaneously, suggesting the potential for synergistic or additive effects. This complexity adds to the challenges of risk characterization. The results obtained from this study highlight the need for occupational hygienists to recognize mycotoxin exposure as a potential occupational hazard, advocating for the implementation of risk management measures to minimize exposure to the lowest feasible level.

## Data Availability

The original contributions presented in the study are included in the article/Supplementary material. Further inquiries can be directed to the corresponding author.

## References

[1] Edite M Freire F Erlan F Izabel M Rondina D. Mycotoxins and their effects on human and animal health. Food Control. (2014) 36:159–65. doi: 10.1016/j.foodcont.2013.08.021, PMID: 39901975

[2] Ostry V Malir F Toman J Grosse Y. Mycotoxins as human carcinogens—the IARC monographs classification. Mycotoxin Res. (2017) 33:65–73. doi: 10.1007/s12550-016-0265-7, PMID: 27888487

[3] Assunção R Viegas S. Mycotoxin exposure and related diseases. Toxins (Basel). (2020) 12:172. doi: 10.3390/toxins12030172, PMID: 32168950 PMC7150930

[4] Mayer S. Occupational exposure to mycotoxins and preventive measures In: Viegas C Pinheiro AC Sabino R Viegas S Brandão J Veríssimo C, editors. Environmental mycology in public health: Fungi and mycotoxins risk assessment and management. United States of America: Elsevier (2016). 325–41.

[5] Ammann HM. Inhalation exposure and toxic effects of mycotoxins In: Li D-W, editor. Biology of microfungi. Switzerland: Springer (2016). 495–523.

[6] Boonen J Malysheva SV Taevernier L Diana Di Mavungu J De Saeger S De Spiegeleer B. Human skin penetration of selected model mycotoxins. Toxicology. (2012) 301:21–32. doi: 10.1016/j.tox.2012.06.012, PMID: 22749975

[7] Viegas S Veiga L Figueiredo P Almeida A Carolino E Viegas C. Assessment of workers’ exposure to aflatoxin B1 in a Portuguese waste industry. Ann Occup Hyg. (2014) 59:173–81. doi: 10.1093/annhyg/meu082 25324565

[8] Viegas S Osteresch B Almeida A Cramer B Humpf H-U Viegas C. Enniatin B and ochratoxin a in the blood serum of workers from the waste management setting. Mycotoxin Res. (2018) 34:85–90. doi: 10.1007/s12550-017-0302-1, PMID: 29185179

[9] Schlosser O Robert S Noyon N. Airborne mycotoxins in waste recycling and recovery facilities: occupational exposure and health risk assessment. Waste Manag. (2020) 105:395–404. doi: 10.1016/j.wasman.2020.02.031, PMID: 32126367

[10] Viegas S Viegas C Martins C Assunção R. Occupational exposure to mycotoxins—different sampling strategies telling a common story regarding occupational studies performed in Portugal (2012–2020). Toxins (Basel). (2020) 12:513. doi: 10.3390/toxins12080513, PMID: 32796626 PMC7472215

[11] Marcelloni AM Pigini D Chiominto A Gioffrè A Paba E. Exposure to airborne mycotoxins: the riskiest working environments and tasks. Ann Work Expo Heal. (2024) 68:19–35. doi: 10.1093/annweh/wxad070, PMID: 38016180 PMC10773202

[12] Viegas S Assunção R Martins C Nunes C Osteresch B Twarużek M. Occupational exposure to mycotoxins in swine production: environmental and biological monitoring approaches. Toxins (Basel). (2019) 11:78. doi: 10.3390/toxins11020078, PMID: 30717100 PMC6410041

[13] Straumfors A Uhlig S Eriksen GS Heldal KK Eduard W Krska R. Mycotoxins and other fungal metabolites in grain dust from Norwegian grain elevators and compound feed mills. World Mycotoxin J. (2015) 8:361–73. doi: 10.3920/WMJ2014.1799

[14] Awuchi CG Ondari EN Nwozo S Odongo GA Eseoghene IJ Twinomuhwezi H. Mycotoxins’ toxicological mechanisms involving humans, livestock and their associated health concerns: a review. Toxins (Basel). (2022) 14:167. doi: 10.3390/toxins14030167, PMID: 35324664 PMC8949390

[15] Eriksen E Graff P Pedersen I Straumfors A Afanou AK. Bioaerosol exposure and in vitro activation of toll-like receptors in a Norwegian waste sorting plant. Saf Health Work. (2022) 13:9–16. doi: 10.1016/j.shaw.2021.09.002, PMID: 35936194 PMC9349000

[16] Eriksen E Afanou AK Madsen AM Straumfors A Graff P. An assessment of occupational exposure to bioaerosols in automated versus manual waste sorting plants. Environ Res. (2023) 218:115040. doi: 10.1016/j.envres.2022.115040, PMID: 36521541

[17] Eriksen E Madsen AM Afanou AK Straumfors A Eiler A Graff P. Occupational exposure to inhalable pathogenic microorganisms in waste sorting. Int J Hyg Environ Health. (2023) 253:114240. doi: 10.1016/j.ijheh.2023.114240, PMID: 37633050

[18] Susitaival P Flyvholm M-A Meding B Kanerva L Lindberg M Svensson Å. Nordic occupational skin questionnaire (NOSQ-2002): a new tool for surveying occupational skin diseases and exposure. Contact Derm. (2003) 49:70–6. doi: 10.1111/j.0105-1873.2003.00159.x, PMID: 14641353

[19] Warth B Sulyok M Berthiller F Schuhmacher R Krska R. New insights into the human metabolism of the *Fusarium mycotoxins* deoxynivalenol and zearalenone. Toxicol Lett. (2013) 220:88–94. doi: 10.1016/j.toxlet.2013.04.012, PMID: 23623764

[20] EFSA (2020). Scientific opinion on Ochratoxin a in food. Parma. Available at: https://www.efsa.europa.eu/sites/default/files/consultation/consultation/CONTAM_3502_draft_opinion_OTA_rev.40.pdf

[21] Apel P Rousselle C Lange R Sissoko F Kolossa-Gehring M Ougier E. Human biomonitoring initiative (HBM4EU)-strategy to derive human biomonitoring guidance values (HBM-GVs) for health risk assessment. Int J Hyg Environ Health. (2020) 230:113622. doi: 10.1016/j.ijheh.2020.113622, PMID: 33045523

[22] Vidal A Claeys L Mengelers M Vanhoorne V Vervaet C Huybrechts B. Humans significantly metabolize and excrete the mycotoxin deoxynivalenol and its modified form deoxynivalenol-3-glucoside within 24 hours. Sci Rep. (2018) 8:5255. doi: 10.1038/s41598-018-23526-9, PMID: 29588479 PMC5869592

[23] Studer-Rohr I Schlatter J Dietrich DR. Kinetic parameters and intraindividual fluctuations of ochratoxin a plasma levels in humans. Arch Toxicol. (2000) 74:499–510. doi: 10.1007/s002040000157, PMID: 11131029

[24] EFSA CONTAM Panel. Risks for animal health related to the presence of zearalenone and its modified forms in feed. EFSA J. (2017) 15:e04851. doi: 10.2903/j.efsa.2017.4851, PMID: 32625539 PMC7009830

[25] EFSA CONTAM Panel. Risks to human and animal health related to the presence of deoxynivalenol and its acetylated and modified forms in food and feed. EFSA J. (2017) 15:e04718. doi: 10.2903/j.efsa.2017.4718, PMID: 32625635 PMC7010102

[26] Apel P Lamkarkach F Lange R Sissoko F David M Rousselle C. Human biomonitoring guidance values (HBM-GVs) for priority substances under the HBM4EU initiative – new values derivation for deltamethrin and cyfluthrin and overall results. Int J Hyg Environ Health. (2023) 248:114097. doi: 10.1016/j.ijheh.2022.114097, PMID: 36577283

[27] EFSA. Management of left-censored data in dietary exposure assessment of chemical substances. EFSA J. (2010) 8:1–96. doi: 10.2903/j.efsa.2010.1557, PMID: 39691501

[28] Martins C Vidal A De Boevre M De Saeger S Nunes C Torres D. Exposure assessment of Portuguese population to multiple mycotoxins: the human biomonitoring approach. Int J Hyg Environ Health. (2019) 222:913–25. doi: 10.1016/j.ijheh.2019.06.010, PMID: 31253542

[29] Viegas C Eriksen E Gomes B Dias M Cervantes R Pena P. Comprehensive assessment of occupational exposure to microbial contamination in waste sorting facilities from Norway. Front Public Health. (2023) 11:1297725. doi: 10.3389/fpubh.2023.1297725 38179569 PMC10766354

[30] Viegas S Viegas C Oppliger A. Occupational exposure to mycotoxins: current knowledge and prospects. Ann Work Expo Heal. (2018) 62:923–41. doi: 10.1093/annweh/wxy070, PMID: 30099513

[31] Viegas S Assunção R Nunes C Osteresch B Twarużek M Kosicki R. Exposure assessment to mycotoxins in a Portuguese fresh bread dough company by using a multi-biomarker approach. Toxins (Basel). (2018) 10:342. doi: 10.3390/toxins10090342, PMID: 30142887 PMC6162618

[32] Ndaw S Jargot D Antoine G Denis F Melin S Robert A. Investigating multi-mycotoxin exposure in occupational settings: a biomonitoring and airborne measurement approach. Toxins (Basel). (2021) 13:54. doi: 10.3390/toxins13010054, PMID: 33450876 PMC7828332

[33] Föllmann W Ali N Blaszkewicz M Degen GH. Biomonitoring of mycotoxins in urine: pilot study in mill workers. J Toxicol Environ Heal - Part A Curr Issues. (2016) 79:1015–25. doi: 10.1080/15287394.2016.1219540, PMID: 27924714

[34] Degen GH. Are we ready to estimate daily ochratoxin a intake based on urinary concentrations? Environ Int. (2016) 97:254–5. doi: 10.1016/j.envint.2015.10.010, PMID: 26602605

[35] Fuchs TC Hewitt P. Biomarkers for drug-induced renal damage and nephrotoxicity—an overview for applied toxicology. AAPS J. (2011) 13:615–31. doi: 10.1208/s12248-011-9301-x, PMID: 21969220 PMC3231866

[36] Drakvik E Altenburger R Aoki Y Backhaus T Bahadori T Barouki R. Statement on advancing the assessment of chemical mixtures and their risks for human health and the environment. Environ Int. (2020) 134:105267. doi: 10.1016/j.envint.2019.105267, PMID: 31704565 PMC6979318

[37] EFSA SC More SJ Bampidis V Benford D Bragard C Hernandez-Jerez A. Guidance document on scientific criteria for grouping chemicals into assessment groups for human risk assessment of combined exposure to multiple chemicals. EFSA J. (2021) 19:e07033. doi: 10.2903/j.efsa.2021.7033, PMID: 34976164 PMC8681880

[38] VKM (2013). Risk assessment of mycotoxins in cereal grain in Norway - opinion of the scientific steering Committee of the Norwegian Scientific Committee for food safety, VKM report 2013: 21. Oslo. pp. 1–287.

[39] Eriksen GS Knutsen HK Sandvik M Brantsæter A-L. Urinary deoxynivalenol as a biomarker of exposure in different age, life stage and dietary practice population groups. Environ Int. (2021) 157:106804. doi: 10.1016/j.envint.2021.106804, PMID: 34352564

[40] Viegas C Twarużek M Dias M Almeida B Carolino E Kosicki R. Assessment of the microbial contamination of mechanical protection gloves used on waste sorting industry: a contribution for the risk characterization. Environ Res. (2020) 189:109881. doi: 10.1016/j.envres.2020.109881, PMID: 32979993

