# Corrigendum: Unraveling the occupational exposure to mycotoxins in a waste management setting: results from a case study in Norway

**DOI:** 10.3389/fpubh.2025.1589437

**Published:** 2025-03-21

**Authors:** Carla Martins, Carla Viegas, Elke Eriksen, Pål Graff, Anani Komlavi Afanou, Anne Straumfors, Magdalena Twarużek, Jan Grajewski, Robert Kosicki, Susana Viegas

**Affiliations:** ^1^NOVA National School of Public Health, Public Health Research Centre, Comprehensive Health Research Center, CHRC, REAL, CCAL, NOVA University Lisbon, Lisbon, Portugal; ^2^H&TRC–Health and Technology Research Center, ESTeSL–Escola Superior de Tecnologia da Saúde, Instituto Politécnico de Lisboa, Lisbon, Portugal; ^3^National Institute of Occupational Health (STAMI), Oslo, Norway; ^4^Department of Physiology and Toxicology, Faculty of Biological Sciences, Kazimierz Wielki University, Bydgoszcz, Poland

**Keywords:** mycotoxins, occupational hygiene, waste management, human biomonitoring, exposure assessment

In the published article, there was an error in affiliation for Elke Eriksen.

Instead of “affiliation 1,2”, it should be “affiliation 3”.

The authors apologize for this error and state that this does not change the scientific conclusions of the article in any way. The original article has been updated.

