# Supplementary material for: Unraveling the occupational exposure to mycotoxins in a waste management setting: results from a case study in Norway
Source: Front Public Health. 2025 Feb 13;13:1536836. doi: 10.3389/fpubh.2025.1536836 (PMC11865241; doi:10.3389/fpubh.2025.1536836)
Supplement: Supplementary file 1 [file Data_Sheet_1.pdf]

Participant	DON (µg/kg bw/day)	ZEN (µg/kg bw/day)	a-ZOL *	β-ZOL*	HT-2** (µg/L)	OTA (µg/kg bw/day)
Outwith control	0.008					
Outwith control	0.010					0.002
Outwith control	0.006					
Outwith control	0.006					0.002
Outwith control	0.001					
Within control	0.043					
Within control	0.020					
Within control						0.006
Within control	0.072					
Within control	0.007					0.006
Within control	0.021					
Within control	0.011					0.006
Within control	0.149					
Within control	0.001					0.002
Within control	0.006					
Within control	0.008					0.031
Within control	0.013					0.002
Within control	0.053					0.018
Within control	0.064					
Worker	0.002					
Worker	0.017					0.038
Worker	0.022					0.006
Worker	0.005					
Worker	0.002					
Worker	0.006					
Worker	0.044	0.001				
Worker	0.068	0.024			0.028	
Worker	0.018	0.053			0.07	
Worker						
Worker	0.068	0.001			0.030	
Worker	0.034	0.001				0.006
Worker	0.061					
Worker	0.041	0.001				
Worker	0.010					
Worker						
Worker	0.036					
Worker	0.002					
Worker	0.019					
Worker	0.033					
Worker	0.022					
Worker	0.009					
Worker	0.019	0.001				0.023
Worker	0.078					
Worker						
Worker	0.012					0.006
Worker	0.009	0.001				
Worker	0.097	0.002				
Worker	0.072					0.026
Worker	0.043					
Worker	0.052					
Worker	0.029					
Worker						

* Concentrations of biomarkers α–ZOL and β-ZOL were included in the estimation of exposure to ZEN for comparison with group TDI (EFSA, 2017). Grey cells indicate that participants presented concentrations of these biomarkers above the limit of detection.

** HT2 - excretion rate not available; results refer to urinary concentration.

µg/kg bw/day = micrograms of mycotoxin per kilogram of body weight per day

Figure S1. Probable Daily Intake estimation for risk characterization purposes. Co-exposure to different mycotoxins, at individual level, is also depicted.
